# Association Between SIRT1, Cytokines, and Metabolic Syndrome in Schizophrenia Patients With Olanzapine or Clozapine Monotherapy

**DOI:** 10.3389/fpsyt.2020.602121

**Published:** 2020-11-25

**Authors:** Xinyu Fang, Lingfang Yu, Dandan Wang, Yan Chen, Yewei Wang, Zenan Wu, Ruimei Liu, Juanjuan Ren, Wei Tang, Chen Zhang

**Affiliations:** ^1^Schizophrenia Program, Shanghai Mental Health Center, Shanghai Jiao Tong University School of Medicine, Shanghai, China; ^2^Affiliated Nanjing Brain Hospital, Nanjing Medical University, Nanjing, China; ^3^The Affiliated Kangning Hospital of Wenzhou Medical University, Wenzhou, China

**Keywords:** schizophrenia, SIRT1, cytokine, metabolic syndrome, olanzapine, clozapine

## Abstract

**Objective:** Previous studies consistently showed the interaction between Sirtuin 1 (SIRT1) and immune inflammation is significantly related to metabolic abnormalities, but their role in the pathogenesis of metabolic syndrome caused by second-generation antipsychotics (SGAs) in schizophrenia patients largely remains unknown. Hence, the present study aimed to fill this gap.

**Methods:** A total of 54 schizophrenia patients with olanzapine or clozapine monotherapy [metabolic syndrome (MetS)/non-MetS patients, 27/27] and 67 healthy subjects were recruited in the present study. The Positive and Negative Syndrome Scale was used, and the plasma levels of SIRT1, interleukin 6 (IL-6), IL-8, IL-10, and tumor necrosis factor α (TNF-α) were measured.

**Results:** The results showed that schizophrenia patients treated with olanzapine or clozapine (both MetS and non-MetS groups) had significantly higher plasma levels of IL-6, IL-10, and TNF-α compared to normal controls (all *P* < 0.05). Moreover, the MetS patients exhibited markedly lower plasma levels of SIRT1 and higher plasma levels of IL-6 than non-MetS patients and normal controls (all *P* < 0.05). However, there were no significant differences in IL-8 levels between groups. Our correlation analysis showed that SIRT1 was significantly correlated with diastolic blood pressure, triglyceride, and high-density lipoprotein cholesterol in schizophrenia patients. The stepwise logistic regression analysis further identified the IL-6 × SIRT1 (β = −0.463, *t* = 10.040, *P* = 0.002) as the influencing factor for the MetS in the patients.

**Conclusion:** Our preliminary findings suggest that SIRT1 interacted with inflammatory cytokines associated with MetS in schizophrenia patients treated with SGA monotherapy.

## Introduction

Schizophrenia is a severe and debilitating psychiatric disorder characterized by a wide range of symptoms, including positive symptoms, negative symptoms, and impaired cognition (1). Antipsychotic drugs have been widely used to treat schizophrenia patients since the advent of chlorpromazine uniformly has alleviated positive symptoms in the 1950s (2). In recent decades, second-generation antipsychotics (SGAs) have been more frequently prescribed for schizophrenia patients profiting from the lower risk of extrapyramidal symptoms and tardive dyskinesia compared to the first-generation antipsychotics (FGAs) (3). Unfortunately, most SGAs, led by clozapine and olanzapine, cause serious metabolic adverse effects including weight gain, abdominal obesity, hyperglycemia, hypertension, and dyslipidemia (4). The Clinical Antipsychotic Trials of Intervention Effectiveness trial reported the prevalence of metabolic syndrome (MetS) to be around 40% in schizophrenia patients based on the National Cholesterol Education Program–Adult Treatment Panel III criteria (NCEP-ATP III) (5). Our recent study supports that schizophrenia patients receiving olanzapine monotherapy more than 2 years had a 44% risk of developing MetS (2). MetS occurring in schizophrenia patients usually leads to several negative outcomes such as poor cognitive function, non-compliance, higher residual psychopathology (6), lower life expectancy, and increased mortality rates (7). Therefore, searching for molecular markers of MetS induced by SGAs is of great significance to elucidate its pathological mechanism.

In the past decade, emerging research has explored the molecular mechanisms underlying the possible processes for SGA-induced metabolic abnormalities. As SGAs act at multiple neurotransmitter receptors, including dopamine D_1_, D_2_, D_3_, and D_4_; adrenergic α1 and α2; serotonin 5HT_2A_ and 5HT_2C_; and muscarinic and histamine H receptors compared to FGAs, the researchers found that these different receptors may be associated with metabolic abnormalities associated with SGAs (8), especially 5HT_2A_, 5HT_2C_, and H receptors, which has an effect on food intake via the hypothalamus (9). In addition, pathways related to altered ghrelin and leptin release, dysfunction of mitochondria, oxidative stress reactions, dysfunctions in the autonomic nervous system activity, altered gut microbiome, and aberrant immune-inflammatory system have also been implicated as important contributing processes leading to MetS with the use of SGAs (2, 10). However, relevant debate and exploration are ongoing, and new molecular markers responsible for the SGAs-caused MetS need to be developed.

Sirtuin 1 (SIRT1) is a highly conserved NAD^+^-dependent protein deacetylase and is widely distributed in the body. Emerging evidence has shown that SIRT1 has important effects on the regulation of lipid and glucose metabolism (11, 12). Early studies indicated that *SIRT1* gene expression in peripheral blood and adipose tissue was significantly diminished in obese subjects compared to control subjects (13), and patients with fatty liver or diabetes also showed reduced plasma levels of SIRT1 (14, 15). In animal models, it was demonstrated that mice with *SIRT1* adipocyte-specific knockout gained weight rapidly, along with glucose intolerance and insulin resistance compared with control mice (16), while the SIRT1 agonist resveratrol could prevent or ameliorate glucose and lipid metabolic disturbance (17). Furthermore, a recent study found oral resveratrol supplementation could improve MetS features in obese patients (18). Taken together, down-regulation of the *SIRT1* may contribute to obesity-associated metabolic abnormalities. However, little is known about the relationship between SIRT1 and SGAs-caused MetS in schizophrenia patients. Interestingly, recent research showed that paliperidone, one of the SGAs, could significantly decrease the expression and production of SIRT1 *in vitro* (19). Thus, the role of SIRT1 on MetS induced by SGAs and its potential mechanism require further investigation.

Chronic and low-grade inflammation is invariably a common feature of the MetS and obesity, where inflammatory signals originate from within visceral adipose tissue in addition to the peripheral system (20, 21). SGAs also potentiate aberrant peripheral levels of inflammatory markers (22, 23), which are associated with their effect on weight gain (22). A study of RNA sequencing revealed that pathways related to the immune inflammatory system were highly enriched with differential expression genes in antipsychotics-caused obesity in patients with schizophrenia (24). Furthermore, our recent work indicated that schizophrenia patients with MetS present an increased expression and production of inflammatory indices (2, 25). This phenomenon has also been supported by other studies showing that altered cytokines levels were significantly associated with the components of MetS induced by SGAs (26–28). In addition, previous clinical observations demonstrated that supplement anti-inflammatory therapy can correct SGAs-caused metabolic abnormalities in schizophrenia patients and mice (29, 30).

Interestingly, previous studies have shown that SIRT1 played a vital role in influencing immune responses (31), and SIRT1 can regulate immune responses directly through deacetylation of some key transcriptional factors or indirectly through metabolic pathways (32). A recent study found that diabetic patients and obese subjects had higher serum IL-6 but lower SIRT1 levels compared to healthy subjects (33). Further evidence from another recent study also showed SIRT1 agonist resveratrol could significantly inhibit the immune inflammation of the body and improve the dysfunction of glycolipid metabolism (32, 34). Thus, altered SIRT1 that interacted with immune inflammation may be associated with MetS caused by SGAs. However, no study to date has explored this issue.

Clozapine and olanzapine are more notorious for causing MetS compared to other SGAs. In light of the abovementioned findings, the present study aimed to investigate the association between SIRT1, inflammatory cytokine levels, and MetS in schizophrenia patients with a long-term olanzapine or clozapine monotherapy and to evaluate if any of such association was mediated independently or interactively in relation to MetS in this population. We hypothesized that the reduced SIRT1 plasma levels may be associated with altered inflammatory cytokines in schizophrenia, and the interaction between SIRT1 and cytokines may contribute to the MetS caused by SGAs in schizophrenia patients.

## Methods

### Participants

A total of 54 schizophrenia patients (27 patients with MetS and 27 patients with non-MetS) with age range from 18 to 50 years were included in the present study. All schizophrenia patients were recruited from Shanghai Mental Health Center and met the following criteria: (1) had been diagnosed with schizophrenia according to the *Diagnostic and Statistical Manual of Mental Disorders, Fourth Edition* (*DSM-IV*) (corresponding to the *International Classification of Diseases, 10th Revision* item F20), diagnoses were made using modified sections of the Structured Clinical Interview for *DSM-IV* disorders by at least 2 experienced psychiatrists; (2) age 18–50 years, Han Chinese; (3) had a junior high school education or above; and (4) patients were receiving clozapine or olanzapine monotherapy for at least 6 months. The exclusion criteria included the following (1) had head trauma with residual effects, neurological disorders; (2) pregnant or breastfeeding; (3) had other diagnosed psychiatric disorder besides schizophrenia or a lifetime substance abuse/dependence disorder; (4) had a recent history of high-grade fever/infection within the past 1 month or any comorbid medical disease that could potentially influence the immune system; and (5) the patients received no drug(s), including statins or anti-diabetes medication, with well-known effects on the study parameters. We also recruited 67 healthy control subjects with age range from 19 to 50 years, who were screened by a specialized psychiatrist using the Structured Clinical Interview for *DSM-IV*-*TR* Axis I Disorders–Patient Edition. All control subjects were self-reported to have no hypertension, hyperglycemia, and hyperlipidemia-related metabolic diseases.

This study was performed in strict accordance with the Declaration of Helsinki and other relevant national and international regulations. All procedures for this study were reviewed and approved by the institutional review boards of the Shanghai Mental Health Center, and written informed consent was obtained from each participant before the commencement of this study.

### Clinical Measurement

All participants were interviewed face-to-face. Basic sociodemographic data such as age, sex, education level, weight, height, body mass index (BMI), and clinical data (age of onset, disease course, drug type) were recorded by trained psychiatrists. The 30-item Positive and Negative Syndrome Scale (PANSS) was assessed by two trained psychiatrists, who had worked at least 5 years in clinical practice. Repeated assessment for the PANSS total score maintained an interrater correlation coefficient >0.8. The three-factor PANSS model was used to assess the psychopathology of schizophrenia patients, consisting of positive symptoms (P), negative symptoms (N), and general psychiatric symptoms (G).

### Anthropometric and Metabolic Parameters

Based on the definition by the NCEP-ATP III, the MetS was diagnosed by any three of the following criteria: (1) a waist circumference ≥90 cm in Chinese men and ≥80 cm in Chinese women; (2) triglyceride (TG) ≥1.7 mmol/L; (3) high-density lipoprotein cholesterol (HDL) <1.0 mmol/L in men and <1.3 mmol/L in women; (4) blood pressure ≥130/85 mmHg; or (5) fasting glucose (GLU) ≥5.6 mmol/L, which is considered as the best MetS criterion for a Chinese population (2).

Blood samples from the patients were collected between 6:00 and 9:00 a.m. following an overnight fast. The serum was separated, aliquoted, and stored at −80°C until used for the assay. Serum fasting GLU, TG, and HDL levels were measured using an automatic Biochemical Analyzer (HITACHI 7170A, Hitachi, Ltd., Tokyo, Japan). Blood pressure including systolic blood pressure and diastolic blood pressure (DBP) was measured using an automated blood pressure machine before the blood collection. The waist circumference in patients was measured between the lower rib margin and the iliac crest, after a normal expiratory breath as described in our previous article (35, 36).

### Plasma SIRT1 and Cytokines Measurement

A total of 10 mL fasting blood samples were collected from 54 patients and 67 healthy controls between 6:00 and 9:00 a.m. The plasma was separated by centrifugation at 3,000 r/min for 15 min at 4°C and stored at −80°C until used for assay. Plasma levels of SIRT1, interleukin 6 (IL-6), IL-8, IL-10, and tumor necrosis factor α (TNF-α) were using enzyme-linked immunosorbent assay kits (RayBiotech, Norcross, GA). The limitation of detection was 7.8 pg/mL for SIRT1, 1.0 pg/mL for SIRT1, and 0.5 pg/mL for IL-6, IL-8, and IL-10. The experiment performers were blind to all the clinical data. The reproducibility of the assay was tested prior to the measurement of the samples, and the interassay coefficient of variation was <5%.

### Data Analysis

The Statistical Package for the Social Sciences version 23.0 was used for data analysis. First, the Student *t*-test or the analysis of variance or the χ^2^ tests were used to compare the differences between groups as appropriate. The analysis of covariance was further performed to control potential confounding factors, such as age, sex, and education levels. The Fisher least significant difference (LSD) was used for multiple comparisons. Second, we correlated the plasma levels of SIRT1 and cytokines in whole schizophrenia patients. Third, Pearson correlation analysis was used to test the correlation between MetS components and SIRT1 or cytokines, and partial correlation analysis was further conducted to control confounding factors. Finally, stepwise logistic regression analysis was performed to explore the links between plasma levels of SIRT1, cytokines, and MetS and their interaction effects in the patients. MetS were selected as dependent variables, whereas altered plasma values (cytokines and SIRT1) between groups, as well as significant interactions between cytokines and SIRT1, were assessed as independent variables. All statistical tests were two-tailed, and statistical significance was set at α ≤ 0.05.

## Results

### Demographic and Clinical Characteristics Among Control Subjects and MetS and Non-MetS Patients With Schizophrenia

Table 1 shows the demographic data of healthy controls and MetS and non-MetS patients with schizophrenia. There were no significant differences in age, sex, and height among those three groups (all *P* > 0.05), but there were differences in education levels, weight, and BMI (*F* = 4.772, *P* = 0.011; *F* = 9.368, *P* < 0.001, *F* = 13.468, *P* < 0.001, respectively). After further LSD correction for multiple comparisons, we found there were no significant differences in BMI and weight between non-MetS patients and normal controls (both *P* > 0.005). Both MetS and non-MetS patients had lower education levels compared to normal controls (*P* = 0.004, *P* = 0.027, respectively), but no difference between MetS and non-MetS patients (*P* > 0.05). For clinical characteristics between Mets and non-MetS patients, the results showed no significant differences in age at onset, family history of mental illness, and duration of monotherapy with olanzapine or clozapine and olanzapine equivalents calculated based on defined daily doses (37) between those two patient groups (all *P* > 0.05). Patients with MetS had a longer disease courses (*t* = 2.662, *P* = 0.010) compared to non-MetS patients. Our results also showed significant differences in PANSS total scores and PANSS-G subscale scores (PANSS total score: *t* = 2.248, *P* = 0.029; PANSS-G: *t* = 2.275, *P* = 0.027), but no differences in PANSS-P, PANSS-N subscale scores between MetS and non-MetS patients (both *P* > 0.05). In addition, schizophrenia patients with MetS had higher waist circumference (*t* = 3.071, *P* = 0.003), DBP (*t* = 2.955, *P* = 0.005), fasting GLU (*t* = 2.108; *P* = 0.040), and TG (*t* = 5.827; *P* < 0.001), but lower serum HDL levels (*t* = 2.306; *P* = 0.025) compared to non-MetS patients.

**Table 1:** Demographic and clinical characteristics among control subjects, MetS and non-MetS patients with schizophrenia.

	**MetS (*N* = 27)**	**non-MetS (*N* = 27)**	**Controls (*N* = 67)**	**F/t/X^**2**^**	***P***	***Post hoc*** **comparisons**		
						**P^**a**^**	**P^**b**^**	**P^**c**^**
Age (year)	37.96 ± 10.56	34.22 ± 9.77	35.21 ± 8.21	1.266	0.286	0.136	0.190	0.637
Sex				0.200	0.905	1.000	0.720	0.720
Male	14	14	32					
Female	13	13	35					
Education (year)	12.00 ± 2.86	12.59 ± 3.05	14.52 ± 3.46	4.772	0.011	0.489	0.004	0.027
Height (cm)	167.19 ± 8.35	169.41 ± 9.04	166.01 ± 8.07	1.731	0.183	0.503	0.324	0.075
Weight (kg)	73.63 ± 12.02	67.22 ± 12.30	62.87 ± 9.97	9.368	<0.001	0.034	<0.001	0.085
BMI (kg/m^2^)	26.05 ± 3.27	23.34 ± 3.14	22.63 ± 2.62	13.468	<0.001	0.001	<0.001	0.286
Age of onset (year)	21.78 ± 5.89	22.78 ± 6.12		0.612	0.544			
Total course (month)	203.07 ± 102.37	133.93 ± 87.99		2.662	0.010			
Family history				0.912	0.340			
No	22	19						
Yes	5	8						
Antipsychotics				3.000	0.083			
Clozapine	12	6						
Olanzapine	15	21						
Olanzapine equivalents (mg)	21.20 ± 9.94	20.00 ± 8.66		0.475	0.637			
Monotherapy duration (week)	55.07 ± 13.28	52.52 ± 15.60		0.648	0.520			
Waist circumference (cm)	91.89 ± 7.72	84.96 ± 8.82		3.071	0.003			
Fasting GLU (mmol/l)	5.80 ± 1.63	5.03 ± 0.96		2.108	0.040			
Fasting TG (mmol/l)	2.37 ± 1.11	1.03 ± 0.44		5.827	<0.001			
Fasting HDL (mmol/l)	1.03 ± 0.22	1.22 ± 0.35		2.306	0.025			
SBP (mm Hg)	117.82 ± 10.06	113.44 ± 9.30		1.657	0.104			
DBP (mm Hg)	78.59 ± 7.77	73.04 ± 5.93		2.955	0.005			
**PANSS**
P	12.96 ± 3.95	12.04 ± 4.13		0.842	0.403			
N	18.82 ± 7.08	16.44 ± 5.15		1.408	0.165			
G	30.78 ± 5.49	27.52 ± 5.03		2.275	0.027			
Total score	62.93 ± 11.56	56.00 ± 11.08		2.248	0.029			
IL-6 (pg/ml)	9.65 ± 2.40	8.13 ± 2.14	5.10 ± 3.17	37.205	<0.001	0.032	<0.001	<0.001
IL-8 (pg/ml)	3.22 ± 1.25	2.93 ± 1.30	2.87 ± 0.93	1.009	0.368	0.331	0.161	0.808
IL-10 (pg/ml)	3.42 ± 0.79	2.96 ± 0.69	1.77 ± 1.38	24.114	<0.001	0.145	<0.001	<0.001
TNF-α (pg/ml)	7.83 ± 2.09	6.78 ± 1.99	3.57 ± 2.93	39.744	<0.001	0.113	<0.001	<0.001
SIRT1 (pg/ml)	35.05 ± 12.28	49.73 ± 14.96	52.23 ± 17.93	11.058	<0.001	0.004	<0.001	1.000

*Data presented as Mean ± SD or N; Olanzapine equivalents were calculated based on defined daily doses (DDDs); BMI, Body Mass Index; IL, Interleukin; TNF, Tumor Necrosis Factor; SIRT1, Sirtuin 1, GLU, glucose; TG, Triglyceride; HDL, High density lipoprotein; SBP, Systolic Blood Pressure; DBP, Diastolic Blood Pressure; PANSS, Positive and Negative Syndrome Scale; P, positive symptoms; N, negative symptoms; G, general psychiatric symptoms*.

*P^a^: MetS vs. non-MetS; P^b^: MetS vs. controls; P^c^: non-MetS vs. controls*.

### Comparisons of Cytokines and SIRT1 Among MetS Patients, Non-MetS Patients, and Control Subjects

**Figure 1:**
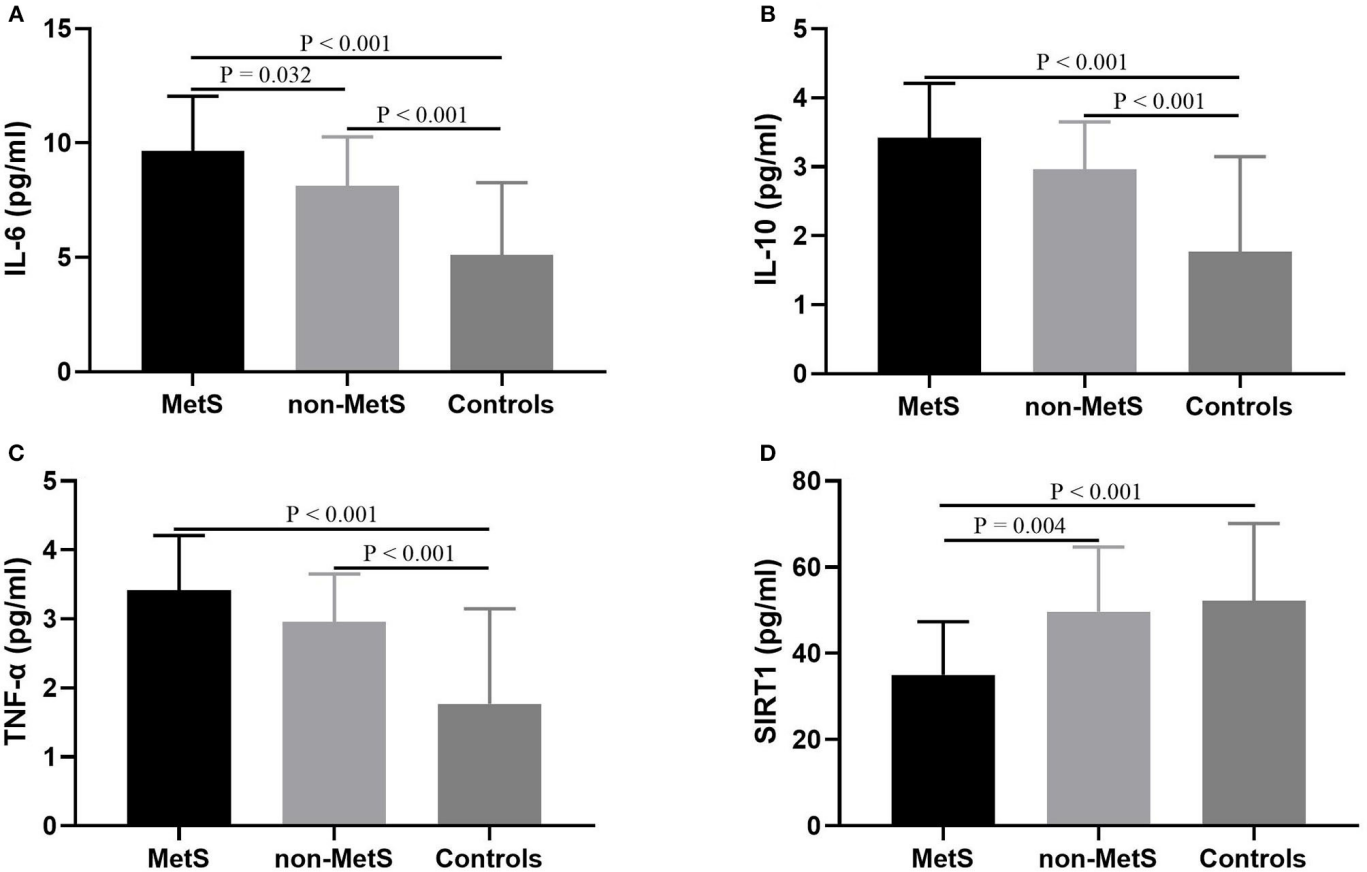
Comparisons of SIRT1 and cytokines levels among MetS patients, non-MetS patients, and control subjects. Each bar represents the mean level of SIRT1 or cytokines. Error bars represent the standard deviation (SD). *P*-value was calculated by adjusting for the characteristics including age, sex, and body mass index. **(A)** Comparison of IL-6 levels among three groups; **(B)** Comparison of IL-10 levels among three groups; **(C)** Comparison of TNF-α levels among three groups; **(D)** Comparison of SIRT1 levels among three groups. MetS, metabolic syndrome; SIRT1, sirtuins; IL, interleukin; TNF, tumor necrosis factor.

As shown in Table 1, plasma levels of IL-6, IL-10, TNF-α, and SIRT1 varied significantly across MetS patients, non-MetS patients, and control subjects (*F* = 37.205, *P* < 0.001; *F* = 24.114, *P* < 0.001; *F* = 39.744, *P* < 0.001; *F* = 11.058, *P* < 0.001, respectively). When age, sex, and education levels were added as covariates, the differences in IL-6, TNF-α, and SIRT1 among those three groups remained significant (*F* = 4.091, *P* = 0.025; *F* = 5.530, *P* = 0.008; *F* = 6.973, *P* = 0.003, respectively), but the difference in IL-10 did not (*P* > 0.05). There was no significant difference in plasma levels of IL-8 among those three groups (*P* < 0.05). After the *post hoc* comparisons using LSD were performed, our results demonstrated that both MetS and non-MetS patients had notably higher IL-6 levels compared to normal controls (both *P* < 0.001), and increased levels of IL-6 were also more pronounced in MetS patients compared to non-MetS patients (*P* = 0.032). In addition, the results indicated that both MetS and non-MetS patients had significantly higher IL-10 and TNF-α compared to control group (both *P* < 0.001), but no significant differences were found between the two patient groups (*P* > 0.05). Interestingly, plasma levels of SIRT1 were markedly lower in MetS patients compared to non-MetS patients (*P* = 0.001) and control (*P* < 0.001), whereas there was no significant difference between non-MetS patients and controls (*P* > 0.05; Figure 1).

### Correlations of SIRT1 and Cytokines in Schizophrenia Patients

In all patients with schizophrenia, our results showed that plasma levels of SIRT1 were negatively correlated with IL-6 (*r* = −0.345, *P* = 0.011), IL-10 (*r* = −0.276, *P* = 0.043), and TNF-α (*r* = −0.393, *P* = 0.003). The correlations between SIRT1 and IL-6 or TNF-α remained significant when partial correlation was performed to control for confounding factors including age, sex, and BMI (*r* = −0.329, *P* = 0.018; *r* = −0.396; *P* = 0.004, respectively), but SIRT1 and IL-10 did not. No significant correlation between plasma levels of SIRT1 and IL-8 were found among all patients (*r* = −0.128, *P* = 0.358).

### Risk Factors for MetS and Its Components in Schizophrenia Patients

**Figure 2:**
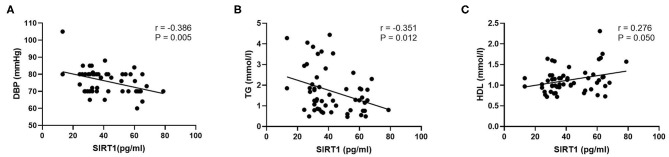
Partial correlation analysis of SIRT1 and DBP, TG, and HDL in schizophrenia patients. *P*-value was calculated by adjusting for the characteristics including age, sex, and body mass index. **(A)** Correlation between SIRT1 and DBP in schizophrenia patients; **(B)** Correlation between SIRT1 and TG in schizophrenia patients; **(C)** Correlation between SIRT1 and HDL in schizophrenia patients. SIRT1, sirtuins; DBP, diastolic blood pressure; TG, triglyceride; HDL, high-density lipoprotein cholesterol.

To identify which MetS components were most strongly associated with SIRT1 or cytokines in schizophrenia patients, the Pearson correlation analysis was performed. The results showed that DBP (*r* = −0.406, *P* = 0.002) and TG (*r* = −0.346, *P* = 0.010) were inversely related with SIRT1 levels in the patients, whereas HDL was positively correlated with SIRT1 levels in the patients (*r* = 0.292, *P* = 0.032). These correlations still remained significant after controlling for age, sex, and BMI (*r* = −0.386, *P* = 0.005; *r* = −0.351, *P* = 0.012; *r* = 0.276, *P* = 0.050, respectively) (Figure 2). Interestingly, our results indicated that IL-6 and IL-10 levels were both correlated with BMI in schizophrenia patients (*r* = 0.233, *P* = 0.010; *r* = 0.204, *P* = 0.025, respectively). However, there were no other significant correlations between SIRT1, IL-6, and IL-10 levels and MetS components in schizophrenia patients (all *P* > 0.05). To further explore the interaction of SIRT1 and cytokines on MetS in schizophrenia patients, the stepwise multiple regression analysis was conducted, with *P*-value criteria of 0.01 and 0.05 for entry and removal, respectively. Our results identified the IL-6 × SIRT1 (β = −0.463, *t* = 10.040, *P* = 0.002) as the influencing factor for the MetS in schizophrenia patients.

## Discussion

To the best of our knowledge, this is the first study that aimed to investigate the association of SIRT1 and MetS caused by SGAs in schizophrenia patients and further to identify the potential interaction of SIRT1 and inflammatory cytokines on the MetS. The main findings in the present study are as follows: (1) schizophrenia patients treated with olanzapine or clozapine (both MetS and non-MetS groups) had significantly higher plasma levels of IL-6, IL-10, and TNF-α compared to normal controls. (2) Moreover, our preliminary study showed that patients exhibited markedly lower plasma levels of SIRT1 and higher plasma levels of IL-6 than non-MetS patients and normal controls. (3) The stepwise logistic regression analysis identified the IL-6 × SIRT1 as the influencing factor for the MetS in schizophrenia patients.

The high risk for the occurrence of MetS in schizophrenia patients may be caused by the combination of multiple factors; coshared genetics and possible risk gene pathway partially explain those comorbidities (38). The *SIRT1* gene is widely recognized as an important role in regulating metabolic status. Ample evidence supports the adipose-specific knockdown of *SIRT1* results in obesity, dyslipidemia, and insulin resistance (39, 40). Moreover, *SIRT1* gene was also reported significantly associated with ambulatory blood pressure level in patients with hypertension (41). Although two early studies explore the relationship between *SIRT1* gene and susceptibility to schizophrenia, and both suggested that altered expression, mutations, and (or) polymorphisms of *SIRT1* might be involved in the pathogenesis of schizophrenia (42), no study to date has explored the relationship between *SIRT1* gene and metabolic dysregulation in schizophrenia patients. Interestingly, increased peripheral levels of SIRT1 were frequently reported to reduce the accumulation of fat and decrease the risk of obesity, hypertension, hyperlipidemia, and diabetes; the low expression of SIRT1 therefore predisposes to the development of MetS (43). Thus, we hypothesized that the high incidence of metabolic dysregulation in schizophrenia patients may be related to the abnormality of SIRT1.

In the present study, we found that MetS patients with SGA monotherapy had markedly lower plasma SIRT1 levels compared to non-MetS patients and healthy controls. Although no study to date has explored the association of peripheral SIRT1 and metabolic abnormalities in schizophrenia, an early study demonstrated that atypical antipsychotics could downregulate the expression of SIRT1 and interfere with the SIRT1 pathway (19). Combined with previous evidence, the findings of the present study suggest that the MetS caused by atypical antipsychotics may be related to its regulation of SIRT1. To further figure out which metabolic components were significantly associated with SIRT1 in schizophrenia patients, we performed correlation analysis and found that SIRT1 was inversely or positively associated with DBP, TG, and HDL, but not GLU. Early study conducted in patients with type 2 diabetes also demonstrated the association of SIRT1 gene and DBP, but not other type 2 diabetes–related traits (41, 44). In addition, previous study shows that patients with arterial stiffness and hypertension had reduced serum levels of SIRT1 compared to normal subjects (44). Qing Gan Zi Shen Tang, as a famous traditional Chinese medicine for its efficacy in treating hypertension, obesity, and hyperlipidemia, and that therapeutic role was recently demonstrated through up-regulation of SIRT1 (19). Hence, pharmacological activation of SIRT1 may be an effective therapeutic strategy for hypertension and hyperlipidemia, and the SIRT1 activator resveratrol further confirmed this hypothesis (45). Although our preliminary study shows no significant correlation between plasma levels of SIRT1 and fasting glucose in schizophrenia patients treated with SGAs, many existing studies to date revealed that reduced SIRT1 is involved in the pathogenesis of hyperglycemia and diabetes (46–49). The non-significant correlation in the present study may be due to our insufficient sample size, and further research with larger sample size is warranted to clarify this.

A promising mechanism underlying the SIRT1 involved in MetS in schizophrenia patients may be associated with the activation of the immune inflammatory system. Substantial evidence supports the activation of inflammatory pathways could disrupt normal metabolism and contribute to the development of metabolic dysfunction (22, 50, 51). In the present study, we found that schizophrenia patients with olanzapine or clozapine monotherapy exhibited increased plasma levels of IL-6, IL-10, and TNF-α, which was also observed in many previous studies (25, 52). Moreover, increased plasma levels of IL-6 and IL-10 were more remarkable in patients with MetS compared to non-MetS patients. Our findings are consistent with some previous studies demonstrating that schizophrenia patients with obesity and MetS displayed markedly elevated levels of proinflammatory factors (2, 29, 53, 54). Interestingly, numerous studies have indicated that SIRT1 plays an important role in immune regulation, and the interaction between SIRT1 and immune inflammation is significantly related to metabolic abnormalities (55, 56). We extended this association to show that the SIRT1 interacting with IL-6 as the influencing factor for the MetS in schizophrenia patients. It is believed that ω-3 polyunsaturated fatty acids (PUFAs) exhibit significant effects on improved insulin sensitivity and obesity, and early study further revealed the effect of ω-3 PUFAs on metabolic is mainly exerted by activating SIRT1 and ensures inhibition of the release of proinflammatory cytokine including IL-6 and TNF-α (57, 58). However, the SIRT1 inhibitor could abrogate the suppression of TNF-α and IL-6 production by ω-3 PUFAs (57). Taken together, decreased SIRT1 may bring out MetS in schizophrenia patients through the activation of inflammatory pathways.

Our preliminary findings must be interpreted with some limitations. The main limitations are obviously the cross-sectional nature of our study, which preclude us to make firm conclusions about the directionality of SIRT1, inflammatory cytokine on MetS caused by SGAs in schizophrenia patients. In the present study, only healthy subjects without MetS were included as controls, whereas MetS subjects without mental illness and not using antipsychotics were not recruited in the present study. The lack of such a control group makes it difficult to distinguish between drug-induced mechanisms for MetS and processes that operate independently of drugs. Furthermore, because of the lack of drug-use data for all the patients before they were treated with olanzapine or clozapine, we could not assess previous treatment and its interference with the present results, which was another limitation of our study. In addition, although olanzapine and clozapine are the two most obvious atypical antipsychotics that cause MetS, the small sample size does not allow our study to be further stratified by drugs. Therefore, a longitudinal study with larger study samples and with best measure to reduce the confounding effect is required to evaluate causal relations between SIRT1, immune inflammation, and MetS in schizophrenia patients.

Taken together, we performed a clinical study to examine the association between SIRT1, inflammatory cytokines, and MetS in schizophrenia patients receiving long-term olanzapine or clozapine monotherapy. Our preliminary study provides evidence suggesting that schizophrenia patients with MetS exhibited reduced plasma SIRT1 but increased inflammatory cytokines levels compared to the non-MetS patients. Moreover, the SIRT1 interacting with IL-6 was significantly responsible for the occurrence of the MetS caused by SGAs in schizophrenia patients. Future longitudinal studies are needed to confirm the role of SIRT1 involved in the pathogenesis of MetS in schizophrenia patients and to further reveal its immune-inflammatory mechanism.

## Data Availability Statement

The raw data supporting the conclusions of this article will be made available by the authors, without undue reservation.

## Ethics Statement

The studies involving human participants were reviewed and approved by Institutional Review Boards of the Shanghai Mental Health Center. The patients/participants provided their written informed consent to participate in this study.

## Author Contributions

XF and CZ: conceptualization and funding acquisition. XF, LY, and DW: methodology, formal analysis, and writing—original draft preparation. XF and LY: software. XF, LY, DW, and CZ: validation. XF, LY, DW, YC, YW, ZW, RL, JR, WT, and CZ: investigation and resources. WT and CZ: data curation and supervision. CZ: writing—review & editing and Project Administration. All authors contributed to the article and approved the submitted version.

## Conflict of Interest

The authors declare that the research was conducted in the absence of any commercial or financial relationships that could be construed as a potential conflict of interest.
